# Brazil between guidelines and reality: the challenges of chronic kidney disease care in Brazil

**DOI:** 10.1590/2175-8239-JBN-2026-E0012en

**Published:** 2026-07-03

**Authors:** Thyago Proença de Moraes, Caio Pellizzari, Maria Fernanda de Aguiar Soares

**Affiliations:** 1 Pontifícia Universidade Católica do Paraná, Curitiba, PR, Brazil.; 2 Nefroclínicas Curitiba, Curitiba, PR, Brazil.

Chronic kidney disease (CKD) is one of the major contemporary public health challenges. Its prevalence has been steadily increasing worldwide, driven by population aging and the growing burden of comorbidities such as diabetes mellitus, hypertension, and obesity, all well-established risk factors for CKD.

In this context, clinical guidelines play a central role by informing strategies for screening, early diagnosis, risk stratification, and timely treatment. In nephrology, the Kidney Disease: Improving Global Outcomes (KDIGO) guidelines have been particularly important in standardizing care through evidencebased recommendations and should help reduce practice variation while improving clinical outcomes. However, translating these recommendations into routine clinical practice remains a substantial challenge.

Barriers such as limited resources, heterogeneity across healthcare systems, unequal access to laboratory testing, restricted availability of medications, and differences in professional training hinder the full implementation of guideline-based care. Understanding how these recommendations are incorporated into real-world practice across different settings is essential to identify gaps and develop locally adapted strategies.

In this issue of the Brazilian Journal of Nephrology, Macedo et al.^1^ present national data on the diagnosis and management of stage 3b CKD by general practitioners and family physicians according to KDIGO recommendations^2^.

In a retrospective cohort study with a three-year follow-up, 211 patients were evaluated, of whom 76% had hypertension and 47% had diabetes. Unfortunately, the findings reveal a substantial gap between theory and practice. Essential tests recommended for this stage of CKD were ordered far less frequently than expected, including assessments for anemia, electrolyte disorders, and mineral and bone metabolism abnormalities. Most concerning, however, was the very low rate of albuminuria testing—a simple and fundamental measure for early diagnosis and risk stratification. Prior to nephrology referral, only 1.4% of patients had a urinary albumin measurement.

**Figure 1:**
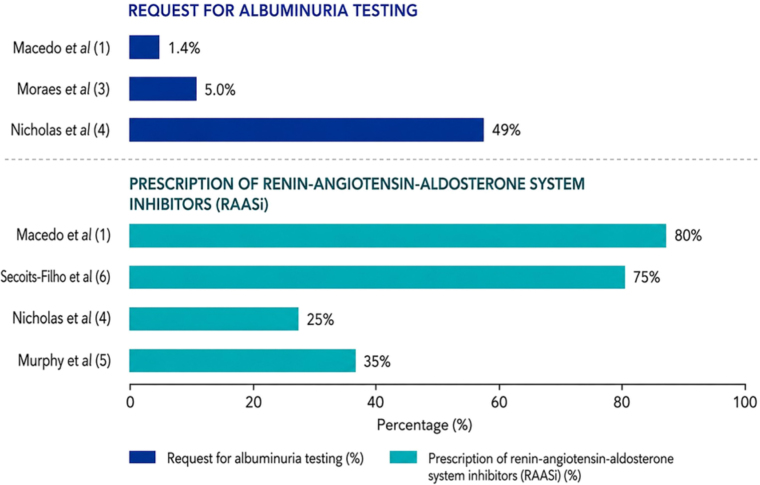
Variation in albuminuria testing and RAAS inhibitor prescription across studies.

These findings are not entirely unexpected (Figure 1). In a recent study also published in our journal, we observed that among patients with diabetes and hypertension but without a previous diagnosis of CKD, albuminuria testing was also markedly underused^3^. Among individuals with renal function stages similar to those reported by Macedo and colleagues, fewer than 5% underwent albuminuria testing during a large national campaign conducted in 2022^3^. Taken together, these data reinforce the difficulty of incorporating simple, inexpensive, and internationally established measures with clear clinical benefit into everyday practice.

An interesting finding, although only briefly explored by the authors, was the high use of renin–angiotensin– aldosterone system inhibitors (RAASi) in this specific population, for whom these agents are formally indicated. This contrasts with both national and international literature, where this drug class has often been underused in CKD, with prescription rates of only 25% in a registry from the United Kingdom and 35% in the United States^4,5^. In Brazil, however, similarly high rates of use of this drug class have previously been reported: data from the CKDOPPS showed that 75% of patients with stage 3b CKD were receiving RAASi therapy^6^. Given that patients with significant albuminuria derive the greatest benefit from this class, these national findings likely reflect greater prescriber familiarity with RAASi therapy and its broad availability in the public healthcare system rather than a targeted implementation of CKD- specific recommendations. An additional point worth noting is that the study period preceded the incorporation of dapagliflozin into the Brazilian public health system; therefore, no relevant information regarding sodium-glucose cotransporter-2 inhibitors (SGLT2i) was available.

The study also revealed a particularly relevant finding: the prolonged delay until the first nephrology consultation in a subgroup recognized as high risk, averaging 20 months and reaching up to 28 months. Equally noteworthy was the absence of any decline in estimated glomerular filtration rate during the first years of follow-up, an unexpected result given that even minimal reductions would generally be anticipated as part of physiological aging. Interpreting the factors behind this observation is not straightforward, but it is difficult to exclude the possibility that the low frequency of essential laboratory testing during this interval may have introduced selection bias.

Finally, several methodological issues should be considered when interpreting these results, including the retrospective design, relatively small sample size, and geographic restriction to a single region. Nevertheless, these limitations do not diminish the relevance of the findings, which are consistent with prior studies demonstrating the inefficiency of the current system in diagnosing CKD.

Closing this gap could not only improve patients’ quality of life and life expectancy but also generate substantial economic benefits for the public healthcare system, potentially creating room for the future incorporation of new medications and other therapies across multiple areas of medicine. The solution, however, is not simple. Appropriately, the authors emphasize that closer integration between nephrology and the specialties that routinely care for these patients is essential. A well-structured collaborative care model, combined with adequate reimbursement, continuing professional education, and formative audit processes, has strong potential to transform the still unsatisfactory scenario we currently face.
