# Supplementary material for: Brazil between guidelines and reality: the challenges of chronic kidney disease care in Brazil
Source: J Bras Nefrol. 2026 Jul 3;48(3):e2026E012. doi: 10.1590/2175-8239-JBN-2026-E0012en (PMC13336663; doi:10.1590/2175-8239-JBN-2026-E0012en)
Supplement: Translation

# Translation: Entre a diretriz e a realidade: os desafios do cuidado da doença renal crônica no Brasil

**DOI:** 10.1590/2175-8239-JBN-2026-E0012pt

**Authors:** Thyago Proença de Moraes, Caio Pellizzari, Maria Fernanda de Aguiar Soares

**Affiliations:** 1 Pontifícia Universidade Católica do Paraná, Curitiba, PR, Brasil.; 2 Nefroclínicas Curitiba, Curitiba, PR, Brasil.

A doença renal crônica (DRC) representa um dos principais desafios contemporâneos em saúde pública, com prevalência crescente em todo o mundo, impulsionada pelo envelhecimento populacional e pelo aumento na prevalência de comorbidades como diabetes mellitus, hipertensão arterial e obesidade, importantes fatores de risco para DRC.

Nesse contexto, diretrizes clínicas assumem papel central ao orientar estratégias de rastreamento, diagnóstico precoce, estratificação de risco e tratamento oportuno. As diretrizes do KDIGO, na nefrologia, exercem esse importante papel na padronização de condutas por meio de suas recomendações e deveriam contribuir para reduzir variações assistenciais e melhorar desfechos clínicos. Entretanto, transformar essas recomendações em prática assistencial rotineira permanece um desafio relevante.

Barreiras como limitações de recursos, heterogeneidade dos sistemas de saúde, acesso desigual a exames laboratoriais, disponibilidade restrita de medicamentos e diferenças na capacitação profissional dificultam a implementação plena das recomendações. Assim, compreender como essas diretrizes são efetivamente incorporadas em diferentes países é essencial para identificar lacunas e adaptar estratégias locais de cuidado.

Nesta edição do *Brazilian Journal of Nephrology*, Macedo et al.^1^ apresentam dados nacionais sobre como é feito o diagnóstico e o manejo da DRC estágio 3b por clínicos gerais e médicos de família, segundo as diretrizes do KDIGO^2^. Em uma coorte retrospectiva com três anos de seguimento, 211 pacientes foram avaliados, dos quais 76% eram hipertensos e 47% diabéticos. Os resultados evidenciam, infelizmente, uma importante distância entre a teoria e a prática. Exames essenciais nesse estágio da DRC foram solicitados com frequência muito abaixo da esperada, incluindo exames para avaliação de anemia, distúrbios eletrolíticos e do metabolismo ósseo-mineral. Porém, o achado mais crítico foi a baixa solicitação da albuminúria, exame simples e fundamental para diagnóstico precoce e estratificação da DRC. Antes da consulta com um nefrologista, somente 1,4% dos pacientes tiveram a albuminúria medida.

Esses achados não são totalmente inesperados (Figura 1). Recentemente, em estudo também publicado em nosso jornal, observamos que, em uma população composta por diabéticos e hipertensos sem diagnóstico prévio de DRC, a solicitação de albuminúria foi muito aquém da necessária^3^. Em indivíduos com estadiamento da função renal semelhantes aos aqui observados por Macedo e colaboradores, menos de 5% tiveram esse exame efetivamente solicitado durante uma ampla campanha nacional realizada em 2022^3^. Em conjunto, esses dados reforçam a dificuldade de incorporar à prática cotidiana medidas simples, baratas e já consolidadas internacionalmente, com reconhecido impacto clínico.

**Figura 1:** Variação na solicitação de albuminúria e na prescrição de inibidores do Sistema Renina-Angiotensina-Aldosterona entre estudos.

Um achado interessante, embora pouco explorado pelos autores, diz respeito ao elevado uso dos inibidores do sistema renina-angiotensina-aldosterona (iSRAA) nessa população específica, que tem indicação formal para essa classe. Esse achado contrasta com a literatura tanto nacional quanto internacional, onde a classe foi subutilizada em pacientes com DRC, sendo prescrita a somente 25% dos indivíduos em um registro inglês e a 35% nos EUA^4,5^. No Brasil, por outro lado, essa elevada prevalência já foi relatada previamente, quando dados do CKDOPPS mostraram que indivíduos com DRC 3b utilizavam um iSRAA em 75% dos casos^6^. Com o conhecimento de que os principais beneficiados da classe são justamente aqueles com albuminúria elevada, não é difícil chegar à conclusão de que esses achados do país sugerem mais uma familiaridade dos prescritores com essa classe terapêutica e sua ampla disponibilidade no sistema público de saúde do que uma aplicação dirigida das recomendações específicas para DRC. Além disso, uma questão adicional que deve ser mencionada é que o momento do estudo foi anterior à inclusão da dapagliflozina no SUS. Desse modo, nenhuma informação relevante sobre a classe dos iSGLT2 foi apresentada.

O estudo revelou um achado particularmente relevante: o prolongado tempo até a primeira consulta com nefrologista em um subgrupo reconhecidamente de alto risco, com média de 20 meses e podendo alcançar 28 meses. Também chama a atenção a ausência de qualquer declínio na taxa de filtração glomerular nos primeiros anos de acompanhamento, resultado pouco esperado, mesmo porque uma redução mínima seria esperada dentro do próprio contexto do envelhecimento fisiológico. Não é simples interpretar os fatores por trás desse achado, mas não podemos descartar, por exemplo, que a baixa realização de exames laboratoriais essenciais durante esse intervalo de tempo possa ter comprometido o resultado por um simples viés de seleção.

Finalmente, é importante considerar questões metodológicas relevantes para a interpretação dos achados. Entre elas estão o delineamento retrospectivo, o tamanho amostral relativamente pequeno e a inclusão de uma população restrita geograficamente a uma região. Entretanto, tais limitações não diminuem a relevância dos achados, que inclusive se alinham com estudos anteriores, como já mencionado, que demonstram a ineficiência do sistema atual no diagnóstico da DRC.

Superar essa lacuna pode não apenas melhorar a qualidade e a expectativa de vida dos pacientes, mas também gerar relevante benefício econômico para o sistema público de saúde, abrindo espaço para a futura incorporação de novos medicamentos e outras terapias em diferentes áreas da medicina. A solução, contudo, não é simples. De forma apropriada, os autores destacam que a maior integração da nefrologia com as especialidades que acompanham esses pacientes na atenção cotidiana exerce papel fundamental. Um matriciamento bem estruturado, associado à remuneração adequada, à educação permanente e à auditoria com caráter formativo, tem grande potencial para transformar o cenário ainda insatisfatório que observamos atualmente.
